# A LODDS-based nomogram for overall and cancer-specific survival in stage III-IV gastric signet ring cell carcinoma

**DOI:** 10.3389/fmolb.2025.1600626

**Published:** 2025-05-14

**Authors:** Cenzhu Wang, Ying Fang, Yuhan Zhang, Shuhan Feng, Rui Hou, Hanfang Fan, Zeyu Wang, Lei Liu, Junli Ding, Junying Xu

**Affiliations:** ^1^ Department of Oncology, The Affiliated Wuxi People’s Hospital of Nanjing Medical University, Wuxi People’s Hospital, Wuxi Medical Center, Nanjing Medical University, Wuxi, China; ^2^ Department of Chemotherapy, Jiangsu Cancer Hospital, The Affiliated Cancer Hospital of Nanjing Medical University, Jiangsu Institute of Cancer Research, Nanjing, China; ^3^ Department of Gastroenterology, The Affiliated Yixing Hospital of Jiangsu University, Yixing, China

**Keywords:** GSRCC, stage III-IV, PLN, LODDS, nomogram

## Abstract

**Objective:**

Gastric cancer is a serious human chronic disease. The gastric signet ring cell carcinoma (GSRCC) is the most-dangerous subtype with several acute complications, including gastrointestinal hemorrhage, gastric perforation, pyloric obstruction and so on. This study aimed to compare the predictive efficiency of positive lymph nodes (PLN) and log odds of positive lymph nodes (LODDS) for survival and to establish a LODDS-based nomogram model in stage III-IV GSRCC.

**Methods:**

Stage III-IV GSRCC patients were acquired between 2015 and 2019 from SEER dataset and the affiliated Yixing hospital of jiangsu university, serving as training and validation datasets respectively. The X-tile software was used to identify cut-off values while their relationship with clinical features was explored by chi-square test. The Kaplan-Meier analysis was applied for survival curve while cox regression analysis was performed for independent risk factors. The nomogram model was built with ROC and calibration curves for verification.

**Results:**

A total of 585 stage III-IV GSRCC patients were included in this study with 536 patients for training and 49 patients for validation. The LODDS showed better predictive efficiency for overall survival (OS) and cancer-specific survival (CSS) than PLN. The LODDS, M stage and chemotherapy status were independent factors for both OS and CSS, with LODDS contribution accounting for 31.47% in OS and 30.39% in CSS. A LODDS-based nomogram was built with accurate efficiency in stage III-IV GSRCC. The 1-year, 2-year, 3-year OS area under curve (AUC) values were 0.755, 0.795, 0.759 for internal and 0.776, 0.756, 0.816 for external verification while 1-year, 2-year, 3-year CSS AUC values were 0.745, 0.803, 0.770 for internal and 0.796, 0.762, 0.820 for external verification.

**Conclusion:**

LODDS is an independent risk factor in stage III-IV GSRCC. The LODDS-based nomogram model showed excellent predictive efficiency, providing a novel insight for early diagnosis and precise therapies of stage III-IV GSRCC.

## Introduction

Gastric cancer is one of the most-common malignant tumors in digestive system, serving as a serious human chronic disease. According to the latest reports, there are 26890 patients occurring gastric cancer in the world, including 16160 males and 10730 females. Meanwhile, there are 10880 patients dying from gastric cancer in the world, including 6,490 males and 4,390 females (Siegel et al., 2024; Lawrence, 1986; Ajani et al., 2022; van Amelsfoort et al., 2022). The risk factors of gastric cancer varies a lot, including high-salt diet, smoking, drinking, HP infection and so on (Smyth et al., 2020; Thrift and El-Serag, 2020; Yeoh and Tan, 2022; Dart, 2024). Gastric signet ring cell carcinoma (GSRCC) is the most dangerous subtype of gastric cancer, approximately accounting for 9.9% in gastric cancer (Humar et al., 2009). GSRCC acquires its name from its typical histologic characteristics, whose cytoplasm is filled with mucus, squeezing the nucleus to the edge and shaping like a ring. As to GSRCC, tumor cells filled with mucus could account for more than 50%, with infiltrating growth and without forming of lumens or glandular ducts. It is reported that GSRCC often happens in young persons with rapid progression, highly invasion and poor survival outcomes, which always progresses into several acute complications, including gastrointestinal hemorrhage, gastric perforation, pyloric obstruction and so on (Zhao et al., 2023; Chen et al., 2023; Li et al., 2022). Compared with stage I-II GSRCC patients, stage III-IV GSRCC patients always suffer from a worse prognosis. As to Li, 4,759 consecutive patients with advanced gastric adenocarcinoma were enrolled for investigation, including 13.9% GSRCC patients. The cumulative 5-year survival rate of advanced GSRCC was 42.4% with more tendency of tumor invasion and lymph node metastasis while Borrmann III-IV served as an independent risk factor for lymph node metastasis (Li et al., 2007). Therefore, it is of great importance to investigate novel biomarkers to realize early diagnosis and precision treatments in stage III-IV GSRCC patients.

Recent researches have investigated the relationship between GSRCC and lymph node metastasis. Yoon SK reported that a 42-year-old man had the early gastric cancer in antral portion with pure signet ring cells confined to the mucosa and widespread perigastric lymph node metastases (Yoon et al., 1987). Besides, Matsuoka K discovered that a 66-year-old man had hilar and mediastinal lymph node metastasis after distal gastrectomy of antral signet ring cell gastric cancer (Matsuoka et al., 2019). Meanwhile, Park JM retrospectively reviewed 215 patients undergoing gastrectomy of early gastric cancer with signet ring cell histology in Korea, finding that 7.9% patients had lymph node metastasis with 1.9% intramucosal cancer and 6% submucosal cancer (Park et al., 2009). What’s more, the data from Korean central cancer registry showed 3.9% mucosal GSRCC patients had regional lymph node metastasis, leading to an increased cancer-related death risk (Jeong et al., 2021). In addition, Pyo JH suggested that three risk factors were associated with lymph node metastasis in mucosa-confined GSRCC, including tumor size larger than 1.7cm, tumors of elevated type and lymphatic-vascular involvement (Pyo et al., 2016). Additionally, Kim YH found that the tubular components served as an important risk factor of lymph node metastasis in GSRCC while Murai K suggested that the absence of double-layer structure was responsible for lymph node metastasis of GSRCC (Kim et al., 2017; Murai et al., 2019).

In this study, we acquired stage III-IV GSRCC patients from 2015 to 2019 years through the Surveillance, Epidemiology and End Results (SEER) dataset and the Chinese hospital named the affiliated Yixing hospital of jiangsu university. Meanwhile, we recognized stage III-IV GSRCC patients from the SEER dataset as the training dataset while we recorded stage III-IV GSRCC patients from Chinese hospital as the validation dataset. The aim of this study was to compare the prediction accuracy between positive lymph nodes (PLN) and log odds of positive lymph nodes (LODDS) for predicting survival outcomes in stage III-IV GSRCC patients. Firstly, we utilized the X-tile software to classify stage III-IV GSRCC patients into three subgroups according to PLN or LODDS respectively. Then, we investigated the relationship between important clinical factors and PLN or LODDS respectively, including age, gender, T stage, N stage, M stage, chemotherapy and so on. Additionally, we explored the predictive efficiency of PLN or LODDS for predicting overall survival (OS) and cancer-specific survival (CSS), discovering that LODDS had the better prediction accuracy than PLN for both OS and CSS in stage III-IV GSRCC patients. What’s more, according to both OS and CSS, we identified the independent risk factors of stage III-IV GSRCC patients, consisting of M stage, chemotherapy and LODDS, which we furtherly constructed the LODDS-based nomogram with great accuracy in both internal and external validation.

## Methods and materials

### Patients acquisition

We acquired stage III-IV GSRCC patients between 2015 and 2019 years from the SEER dataset, which served as the training dataset. Meanwhile, we enrolled stage III-IV GSRCC patients between 2015 and 2019 years from the Chinese hospital named the affiliated Yixing hospital of jiangsu university, which worked as the validation dataset. The inclusion criteria were shown as follows: (1) the primary site of malignant tumor was stomach; (2) the pathological diagnosis of malignant tumor was gastric signet ring cell carcinoma; (3) the TNM stage of malignant tumor was stage III or stage IV. Besides, the exclusion criteria were presented as follows: (1) non-primary or metastatic malignant tumor; (2) the clinical features or survival data of patients were missed. Finally, the number of 536 stage III-IV GSRCC patients from the SEER dataset were screened as the training dataset while the number of 49 stage III-IV GSRCC patients from the Chinese hospital were collected as the validation dataset.

### Ethics and research endpoints

The data of patients from the SEER dataset (https://seerdataaccess.cancer.gov/seer-data-access) was acquired in public. The data of patients from the Chinese hospital was acquired according to the ethics committees of the affiliated Yixing hospital of jiangsu university (IRB Approval No. 2023-140). This study was conducted according to the Helsinki Declaration. The primary endpoint of this study was cancer-specific survival (CSS), which meant the time from GSRCC diagnosis to death specifically due to GSRCC. Meanwhile, the secondary endpoint of this study was overall survival (OS), which meant the time from GSRCC diagnosis to death due to any cause. The survival outcomes of enrolled patients were followed up by telephone.

### Clinical variables and cut-off value identification

The clinical variables of stage III-IV GSRCC patients consisted of age, gender, T stage, N stage, M stage and chemotherapy status. The cut-off values of PLN or LODDS in stage III-IV GSRCC patients were identified by the X-tile software (https://medicine.yale.edu/lab/rimm/research/software/). According to the cut-off values of PLN or LODDS, stage III-IV GSRCC patients were classified into three groups for further investigation, including low, mid and high groups.

### Statistical analysis

We applied the pearson’s chi-square test or fisher’s exact probability test to investigate the relationship between clinical features and research factors in stage III-IV GSRCC patients, including PLN and LODDS. Besides, we performed the Kaplan-Meier method to present the survival curve of OS and CSS in stage III-IV GSRCC patients. In addition, we used the Cox proportional hazards model to identify the independent risk factors of stage III-IV GSRCC patients. Meanwhile, we established a nomogram model based on the independent risk factors of stage III-IV GSRCC patients. The predictive efficiency of nomogram model was assessed in both internal and external validation via the receiver operating characteristic curves (ROC) and calibration curves. The statistical analysis was conducted by SPSS 21.0 and R 4.3.1 software. We applied SPSS software to perform the pearson’s chi-square test or fisher’s exact probability test, Cox proportional hazards model while we used R software to conduct survival analysis, establishment and verification of nomogram model. The relevant R packages included “survival”, “survminer”, “regplot”, “timeROC” and “rms” packages. The P < 0.05 was recognized as significant.

## Results

### Identification of best cut-off values of PLN and LODDS

**FIGURE 1:**
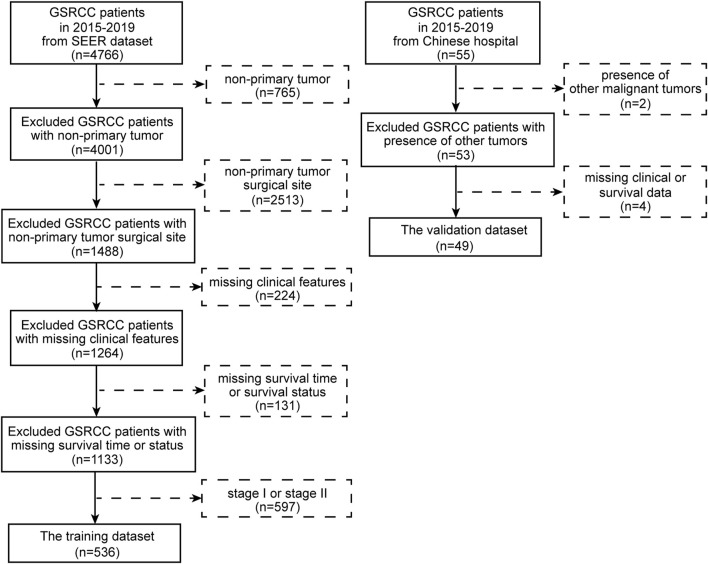
The flowchart of the study.

**FIGURE 2:**
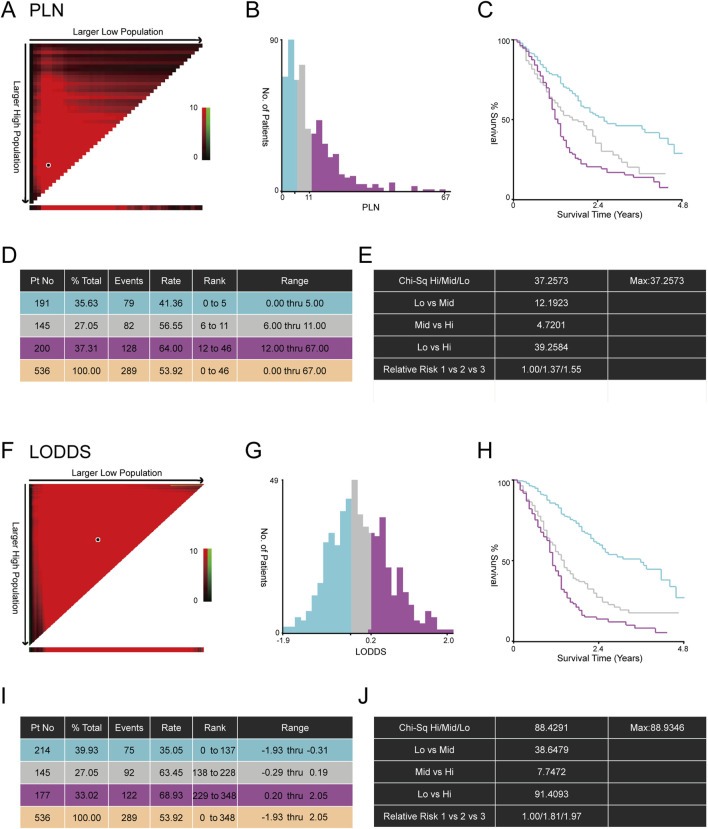
The best cut-off values of PLN and LODDS in stage III-IV GSRCC patients. **(A)** The correlation plot of PLN in stage III-IV GSRCC patients; **(B)** The histogram showing three groups of stage III-IV GSRCC patients according to PLN; **(C)** The Kaplan-Meier survival curve showing three groups of stage III-IV GSRCC patients according to PLN; **(D,E)** Identification of best cut-off values of PLN in stage III-IV GSRCC patients via X-tile software; **(F)** The correlation plot of LODDS in stage III-IV GSRCC patients; **(G)** The histogram showing three groups of stage III-IV GSRCC patients according to LODDS; **(H)** The Kaplan-Meier survival curve showing three groups of stage III-IV GSRCC patients according to LODDS; **(I,J)** Identification of best cut-off values of LODDS in stage III-IV GSRCC patients via X-tile software.

In total, according to the flowchart, 536 stage III-IV GSRCC patients from the SEER dataset were enrolled as the training dataset while 49 stage III-IV GSRCC patients from the Chinese hospital were enrolled as the validation dataset (Figure 1). In addition, we utilized the X-tile software to identify best cut-off values of PLN and LODDS in stage III-IV GSRCC patients in the training dataset. According to PLN, stage III-IV GSRCC patients were classified into three groups: low PLN group (L-PLN, PLN<6), mid PLN group (M-PLN, 6≤PLN≤11) and high PLN group (H-PLN, PLN>11) (Figures 2A–E). Meanwhile, based on LODDS, stage III-IV GSRCC patients were classified into three groups: low LODDS group (L-LODDS, LODDS <−0.29), mid LODDS group (M-LODDS, −0.29≤LODDS≤0.19) and high LODDS group (H-LODDS, LODDS>0.19) (Figures 2F–J).

### Relationship between clinical features and PLN、LODDS in stage III-IV GSRCC patients

We applied the pearson’s chi-square test or fisher’s exact probability test to explore the relationship between clinical features and PLN、LODDS in stage III-IV GSRCC patients, including age, gender, T stage, N stage, M stage and chemotherapy status. According to PLN, the SEER dataset (training dataset) consisted of 191 low-PLN patients, 145 mid-PLN patients and 200 high-PLN patients while the Chinese dataset (validation dataset) consisted of 16 low-PLN patients, 16 mid-PLN patients and 17 high-PLN patients. In the training dataset, PLN was related with T stage and N stage (Table 1).

**TABLE 1:** Relationship between clinical features and PLN in stage III-IV GSRCC patients.

Variable	The SEER dataset				The Chinese dataset			
	Low-PLN (N = 191)	Mid-PLN (N = 145)	High-PLN (N = 200)	*P* Value	Low-PLN (N = 16)	Mid-PLN (N = 16)	High-PLN (N = 17)	*P* Value
Age				0.567				0.406
≤60	94 (49.21)	75 (51.72)	92 (46.00)		3 (18.75)	5 (31.25)	7 (41.18)	
>60	97 (50.79)	70 (48.28)	108 (54.00)		13 (81.25)	11 (68.75)	10 (58.82)	
Gender				0.783				0.269
Male	89 (46.60)	73 (50.34)	95 (47.50)		14 (87.50)	10 (62.50)	13 (76.47)	
Female	102 (53.40)	72 (49.66)	105 (52.50)		2 (12.50)	6 (37.50)	4 (23.53)	
T stage				0.002**				0.204
T1-T2	2 (1.05)	10 (6.90)	3 (1.50)		0 (0.00)	2 (12.50)	0 (0.00)	
T3-T4	189 (98.95)	135 (93.10)	197 (98.50)		16 (100.00)	14 (87.50)	17 (100.00)	
N stage				<0.001***				na
N0	13 (6.81)	0 (0.00)	0 (0.00)		0 (0.00)	0 (0.00)	0 (0.00)	
N1-N3	178 (93.19)	145 (100.00)	200 (100.00)		16 (100.00)	16 (100.00)	17 (100.00)	
M stage				0.555				0.177
M0	161 (84.29)	127 (87.59)	167 (83.50)		13 (81.25)	14 (87.50)	10 (58.82)	
M1	30 (15.71)	18 (12.41)	33 (16.50)		3 (18.75)	2 (12.50)	7 (41.18)	
Chemotherapy				0.101				0.537
No	28 (14.66)	34 (23.45)	42 (21.00)		1 (6.25)	1 (6.25)	0 (0.00)	
Yes	163 (85.34)	111 (76.55)	158 (79.00)		15 (93.75)	15 (93.75)	17 (100.00)	

The significant P values were highlighted in bold.

Meanwhile, according to LODDS, the SEER dataset (training dataset) consisted of 214 low-LODDS patients, 145 mid-LODDS patients and 177 high-LODDS patients while the Chinese dataset (validation dataset) consisted of 19 low-LODDS patients, 8 mid-LODDS patients and 22 high-LODDS patients. In the training dataset, LODDS was associated with N stage, M stage and chemotherapy status (Table 2).

**TABLE 2:** Relationship between clinical features and LODDS in stage III-IV GSRCC patients.

Variable	The SEER dataset				The Chinese dataset			
	Low-LODDS (N = 214)	Mid-LODDS (N = 145)	High-LODDS (N = 177)	*P* Value	Low-LODDS (N = 19)	Mid-LODDS (N = 8)	High-LODDS (N = 22)	*P* Value
Age				0.348				0.731
≤60	112 (52.34)	65 (44.83)	84 (47.46)		5 (26.32)	2 (25.00)	8 (36.36)	
>60	102 (47.66)	80 (55.17)	93 (52.54)		14 (73.68)	6 (75.00)	14 (63.64)	
Gender				0.540				0.184
Male	107 (50.00)	64 (44.14)	86 (48.59)		16 (84.21)	4 (50.00)	17 (77.27)	
Female	107 (50.00)	81 (55.86)	91 (51.41)		3 (15.79)	4 (50.00)	5 (22.73)	
T stage				0.053				0.153
T1-T2	8 (3.74)	6 (4.14)	1 (0.56)		1 (5.26)	1 (12.50)	0 (0.00)	
T3-T4	206 (96.26)	139 (95.86)	176 (99.44)		18 (94.74)	7 (87.50)	22 (100.00)	
N stage				<0.001***				na
N0	13 (6.07)	0 (0.00)	0 (0.00)		0 (0.00)	0 (0.00)	0 (0.00)	
N1-N3	201 (93.93)	145 (100.00)	177 (100.00)		19 (100.00)	8 (100.00)	22 (100.00)	
M stage				0.035*				0.325
M0	180 (84.11)	132 (91.03)	143 (80.79)		16 (84.21)	7 (87.50)	14 (63.64)	
M1	34 (15.89)	13 (8.97)	34 (19.21)		3 (15.79)	1 (12.50)	8 (36.36)	
Chemotherapy				<0.001***				0.153
No	23 (10.75)	32 (22.07)	49 (27.68)		1 (5.26)	1 (12.50)	0 (0.00)	
Yes	191 (89.25)	113 (77.93)	128 (72.32)		18 (94.74)	7 (87.50)	22 (100.00)	

The significant P values were highlighted in bold.

### Kaplan-Meier survival analysis of PLN and LODDS in stage III-IV GSRCC patients

**FIGURE 3:**
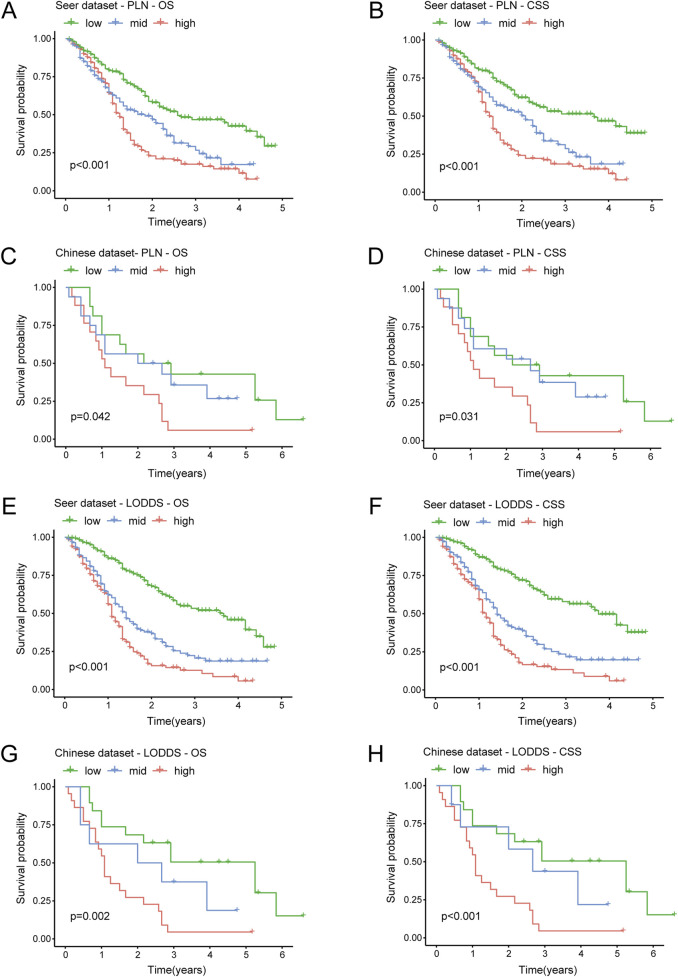
The Kaplan-Meier analysis of PLN and LODDS in stage III-IV GSRCC patients. **(A,B)** The survival analysis of PLN for OS and CSS in the SEER dataset in stage III-IV GSRCC patients; **(C,D)** The survival analysis of PLN for OS and CSS in the Chinese dataset in stage III-IV GSRCC patients; **(E,F)** The survival analysis of LODDS for OS and CSS in the SEER dataset in stage III-IV GSRCC patients; **(G,H)** The survival analysis of LODDS for OS and CSS in the Chinese dataset in stage III-IV GSRCC patients.

We performed the Kaplan-Meier survival analysis to explore the predictive efficiency of PLN and LODDS in stage III-IV GSRCC patients for survival outcomes, including OS and CSS. According to PLN in the SEER dataset (training dataset), low-PLN patients had best survival outcomes, mid-PLN patients had moderate survival outcomes while high-PLN patients had worst survival outcomes, respectively in OS (P < 0.001) and CSS (P < 0.001) in stage III-IV GSRCC patients (Figures 3A,B). Besides, based on PLN in the Chinese dataset (validation dataset), low-PLN patients had best survival outcomes, mid-PLN patients had moderate survival outcomes while high-PLN patients had worst survival outcomes, respectively in OS (P = 0.042) and CSS (P = 0.031) in stage III-IV GSRCC patients (Figures 3C,D).

In addition, as to LODDS in the SEER dataset (training dataset), low-LODDS patients had best survival outcomes, mid-LODDS patients had moderate survival outcomes while high-LODDS patients had worst survival outcomes, respectively in OS (P < 0.001) and CSS (P < 0.001) in stage III-IV GSRCC patients (Figures 3E,F). What’s more, as to LODDS in the Chinese dataset (validation dataset), low-LODDS patients had best survival outcomes, mid-LODDS patients had moderate survival outcomes while high-LODDS patients had worst survival outcomes, respectively in OS (P = 0.002) and CSS (P < 0.001) in stage III-IV GSRCC patients (Figures 3G,H). In summary, the survival predictive efficiency of LODDS showed more accurate performance than PLN in stage III-IV GSRCC patients, especially in the Chinese dataset.

### Cox regression and contribution analysis in stage III-IV GSRCC patients

**FIGURE 4:**
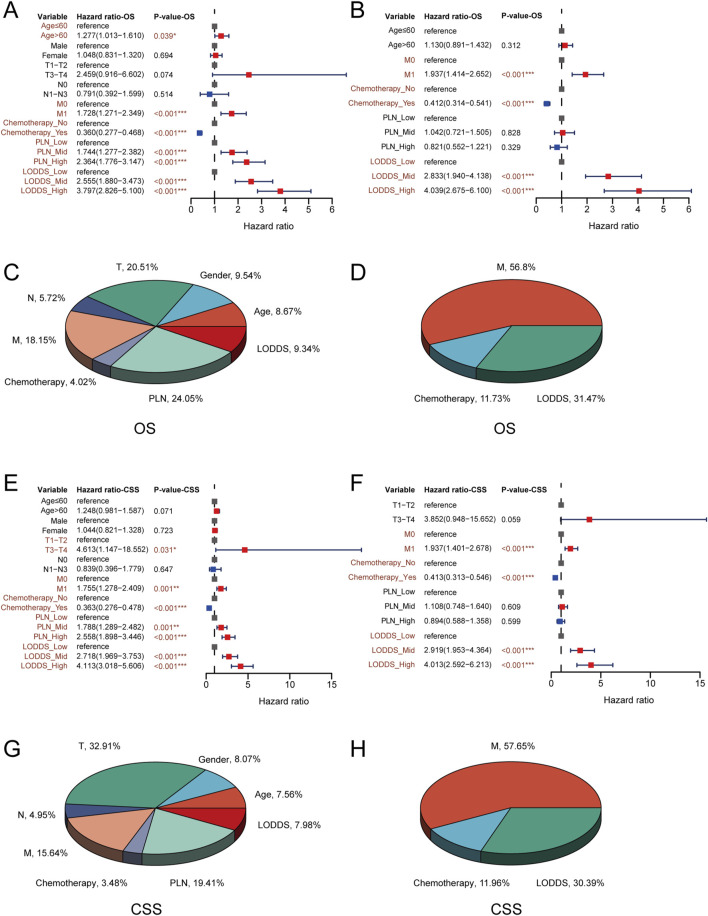
The cox regression and contribution analysis in stage III-IV GSRCC patients. **(A,B)** The univariate and multivariate cox regression analysis of OS in stage III-IV GSRCC patients; **(C,D)** The OS contribution analysis of clinical features and independent factors in stage III-IV GSRCC patients; **(E,F)** The univariate and multivariate cox regression analysis of CSS in stage III-IV GSRCC patients; **(G,H)** The CSS contribution analysis of clinical features and independent factors in stage III-IV GSRCC patients.

We used univariate and multivariate cox regression analysis to identify independent risk factors of OS and CSS in stage III-IV GSRCC patients. As to OS, the univariate cox regression analysis indicated that age, M stage, chemotherapy status, PLN and LODDS were risk factors in stage III-IV GSRCC patients (Figure 4A). What’s more, we included those risk factors into multivariate cox regression analysis, finding that M stage, chemotherapy status and LODDS served as the independent risk factors in stage III-IV GSRCC patients (Figure 4B). Meanwhile, the OS contribution analysis of clinical features and independent risk factors were presented via pie chart in stage III-IV GSRCC patients (Figures 4C,D). As to the independent risk factors of OS in stage III-IV GSRCC patients, M stage accounted for 56.8%, LODDS accounted for 31.47% while chemotherapy status accounted for 11.73%.

With regards to CSS, the univariate cox regression analysis suggested that T stage, M stage, chemotherapy status, PLN and LODDS were risk factors in stage III-IV GSRCC patients (Figure 4E). What’s more, we included those risk factors into multivariate cox regression analysis, discovering that M stage, chemotherapy status and LODDS worked as the independent risk factors in stage III-IV GSRCC patients (Figure 4F). Meanwhile, the CSS contribution analysis of clinical features and independent risk factors were presented via pie chart in stage III-IV GSRCC patients (Figures 4G,H). As to the independent risk factors of CSS in stage III-IV GSRCC patients, M stage accounted for 57.65%, LODDS accounted for 30.39% while chemotherapy status accounted for 11.96%. In summary, according to both OS and CSS, M stage, chemotherapy status and LODDS were identified as the independent risk factors in stage III-IV GSRCC patients (Table 3) (Table 4).

**TABLE 3:** The univariate and multivariate cox regression analysis of OS in stage III-IV GSRCC patients.

Variable	SEER database - OS			
	Univariate		Multivariate	
	HR (95% CI)	P Value	HR (95% CI)	P Value
Age				
≤60	1		1	
>60	1.277 (1.013–1.610)	0.039*	1.130 (0.891–1.432)	0.312
Gender				
Male	1			
Female	1.048 (0.831–1.320)	0.694		
T stage				
T1-T2	1			
T3-T4	2.459 (0.916–6.602)	0.074		
N stage				
N0	1			
N1-N3	0.791 (0.392–1.599)	0.514		
M stage				
M0	1		1	
M1	1.728 (1.271–2.349)	<0.001***	1.937 (1.414–2.652)	<0.001***
Chemotherapy				
No	1		1	
Yes	0.360 (0.277–0.468)	<0.001***	0.412 (0.314–0.541)	<0.001***
PLN				
low	1		1	
mid	1.744 (1.277–2.382)	<0.001***	1.042 (0.721–1.505)	0.828
high	2.364 (1.776–3.147)	<0.001***	0.821 (0.552–1.221)	0.329
LODDS				
low	1		1	
mid	2.555 (1.880–3.473)	<0.001***	2.833 (1.940–4.138)	<0.001***
high	3.797 (2.826–5.100)	<0.001***	4.039 (2.675–6.100)	<0.001***

The significant P values were highlighted in bold.

**TABLE 4:** The univariate and multivariate cox regression analysis of CSS in stage III-IV GSRCC patients.

Variable	SEER database - CSS			
	Univariate		Multivariate	
	HR (95% CI)	P Value	HR (95% CI)	P Value
Age				
≤60	1			
>60	1.248 (0.981–1.587)	0.071		
Gender				
Male	1			
Female	1.044 (0.821–1.328)	0.723		
T stage				
T1-T2	1		1	
T3-T4	4.613 (1.147–18.552)	0.031*	3.852 (0.948–15.652)	0.059
N stage				
N0	1			
N1-N3	0.839 (0.396–1.779)	0.647		
M stage				
M0	1		1	
M1	1.755 (1.278–2.409)	0.001**	1.937 (1.401–2.678)	<0.001***
Chemotherapy				
No	1		1	
Yes	0.363 (0.276–0.478)	<0.001***	0.413 (0.313–0.546)	<0.001***
PLN				
low	1		1	
mid	1.788 (1.289–2.482)	0.001**	1.108 (0.748–1.640)	0.609
high	2.558 (1.898–3.446)	<0.001***	0.894 (0.588–1.358)	0.599
LODDS				
low	1		1	
mid	2.718 (1.969–3.753)	<0.001***	2.919 (1.953–4.364)	<0.001***
high	4.113 (3.018–5.606)	<0.001***	4.013 (2.592–6.213)	<0.001***

The significant P values were highlighted in bold.

### Establishment and verification of LODDS-based nomogram in stage III-IV GSRCC patients

**FIGURE 5:**
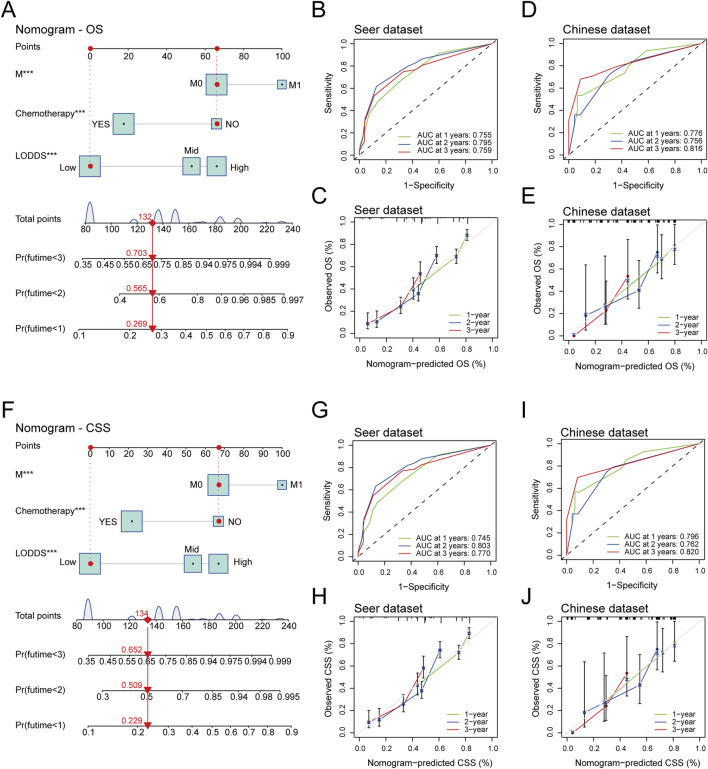
The establishment and verification of LODDS-based nomogram in stage III-IV GSRCC patients. **(A)** The construction of the LODDS-based nomogram for OS in stage III-IV GSRCC patients; **(B,C)** The ROC curve and the calibration curve of the LODDS-based nomogram for OS in the internal verification; **(D,E)** The ROC curve and the calibration curve of the LODDS-based nomogram for OS in the external verification; **(F)** The construction of the LODDS-based nomogram for CSS in stage III-IV GSRCC patients; **(G,H)** The ROC curve and the calibration curve of the LODDS-based nomogram for CSS in the internal verification; **(I,J)** The ROC curve and the calibration curve of the LODDS-based nomogram for CSS in the external verification.

We constructed a LODDS-based nomogram for OS in stage III-IV GSRCC patients in the SEER dataset (training dataset), including independent risk factors of M stage, chemotherapy status and LODDS (Figure 5A). In addition, we utilized the ROC curve and calibration curve to conduct internal and external verification. As to the internal verification, the ROC curve indicated that AUC values of OS for 1-year, 2-year and 3-year were 0.755, 0.795 and 0.759 respectively while the calibration curves of OS for 1-year, 2-year and 3-year were close to the 45-degree diagonal line (Figures 5B,C). Besides, as to the external verification, the ROC curve indicated that AUC values of OS for 1-year, 2-year and 3-year were 0.776, 0.756 and 0.816 respectively while the calibration curves of OS for 1-year, 2-year and 3-year were close to the 45-degree diagonal line (Figures 5D,E).

Meanwhile, we established a LODDS-based nomogram for CSS in stage III-IV GSRCC patients in the SEER dataset (training dataset), including independent risk factors of M stage, chemotherapy status and LODDS (Figure 5F). With regards to the internal verification, the ROC curve indicated that AUC values of CSS for 1-year, 2-year and 3-year were 0.745, 0.803 and 0.770 respectively while the calibration curves of CSS for 1-year, 2-year and 3-year were close to the 45-degree diagonal line (Figures 5G,H). Additionally, based on the external verification, the ROC curve indicated that AUC values of CSS for 1-year, 2-year and 3-year were 0.796, 0.762 and 0.820 respectively while the calibration curves of CSS for 1-year, 2-year and 3-year were close to the 45-degree diagonal line (Figures 5I,J). In summary, the LODDS-based nomogram had a great predictive efficiency for both OS and CSS in stage III-IV GSRCC patients.

## Discussion

Gastric cancer is a common digestive malignant tumor with high incidence and mortality rates (Lochhead and El-Omar, 2008; Zhao et al., 2020; Zhang and Ge, 2013). The GSRCC is the most serious subtype of gastric cancer. Patients with GSRCC always suffer from a bad prognosis, bearing the burden of rapid distant metastases and poor survival outcomes (Silverman et al., 2023). Therefore, it is of great significance to search for novel biomarkers in GSRCC, providing guidance for early diagnosis and precision treatments of GSRCC. Recent researches reported that there were critical association between lymph node metastasis and GSRCC prognosis. This study was designed to compare the predictive efficiency of novel biomarkers associated with lymph node metastasis in stage III-IV GSRCC patients, including PLN and LODDS, discovering that LODDS had a better predictive accuracy than PLN for predicting OS and CSS in stage III-IV GSRCC patients.

In this study, we downloaded stage III-IV GSRCC patients from 2015 to 2019 years through the SEER dataset as the training dataset while we acquired stage III-IV GSRCC patients from 2015 to 2019 years via the Chinese hospital named the affiliated Yixing hospital of jiangsu university as the validation dataset. By using the X-tile software, stage III-IV GSRCC patients were classified into low, mid and high subgroups according to PLN or LODDS respectively. Based on the chi-square test, we found that the PLN was related with T stage, N stage while the LODDS was associated with N stage, M stage and chemotherapy status. In addition, compared with PLN, LODDS presented a better predictive efficiency for predicting survival outcomes of stage III-IV GSRCC, including OS and CSS. What’s more, compared with PLN, LODDS served as an independent risk factor for both OS and CSS of stage III-IV GSRCC patients. Meanwhile, we built the nomogram with independent risk factors of stage III-IV GSRCC patients, consisting of M stage, chemotherapy and LODDS, which showed great predictive efficiency in both SEER dataset and Chinese hospital.

Recently, some researches have discovered the potential relationship between LODDS and other malignant tumors. Liu established a model for predicting CSS of ovarian clear cell carcinoma with four important clinical factors, including age, T stage, stage and LODDS value, which was verified in both SEER dataset and the first affiliated hospital of Dalian medical university (Liu et al., 2024). Besides, He acquired resectable non-small cell lung cancer with N1-N2 stage from both SEER dataset and the first affiliated hospital of zhejiang university school of medicine, finding that nomogram of number of positive lymph nodes (NPLN) and LODDS could serve as an accurate prediction tool (He et al., 2024). What’s more, Gao identified LODDS as a better lymph node assessment tool for predicting survival outcomes in small cell lung cancer patients (Gao et al., 2024). Meanwhile, Han built a LODDS-related nomogram model in advanced colorectal cancer patients receiving neoadjuvant therapy, which showed the great efficiency in SEER dataset and Tangdu hospital (Han et al., 2024). In addition, Zhang enrolled 663 consecutive patients diagnosed stage III colon cancer from Sun Yat-sen university cancer center and Longyan first affiliated hospital of fujian medical university, discovering that the LODDS-based predictive model could provide guidance for postoperative adjuvant chemotherapy duration in stage III colon cancer (Han et al., 2024). As to Cao, Cao explored the N1 stage subgroup of differentiated thyroid cancer, finding that positive lymph node numbers (PLNMs) could act as a more suitable predictive tool than metastatic lymph node ratio (MLNR) and LODDS (Cao et al., 2024). According to Resenda V, the minimum of 20 lymph nodes was acquired for assessment in ampullary cancer patients undergoing pancreatoduodenectomy, suggesting that LODDS had the highest prognostic accuracy (Resende et al., 2024). Additionally, Salari A presented that LODDS could work as an independent predictor for overall survival in patients with urothelial bladder cancer undergoing radical cystectomy (Salari et al., 2023).

However, there are still some limitations in this study for further investigation. On the one hand, the clinical features of enrolled stage III-IV GSRCC patients from SEER dataset or Chinese hospital were limited with several important clinical features missing. We should supplement other significant clinical features in the future, for example, radiotherapy, immunotherapy, targeted therapy and so on. On the other hand, this study acquired stage III-IV GSRCC patients from single-center Chinese hospital as the validation dataset, which should be expanded into multi-center Chinese hospitals for widespread verification in the future. Besides, this study focused on the number of positive lymph nodes without paying attention to the locations of lymph nodes. According to Marrelli D, D2 plus lymphadenectomy after chemotherapy could improve the survival outcomes of locally advanced or oligometastatic gastric cancer patients (Salari et al., 2023). Therefore, we should focus on special locations of positive lymph nodes in the future. In addition, we should investigate the function of LODDS in other subtypes of gastric cancer, exploring the universality of LODDS in gastric cancer.

## Conclusions

In summary, LODDS was recognized as an independent risk factor in stage III-IV GSRCC patients for predicting OS and CSS. A LODDS-based nomogram model was constructed for predicting OS and CSS in stage III-IV GSRCC with great predictive efficiencies, which may provide novel insights for early diagnosis and precision treatment in stage III-IV GSRCC.

## Data Availability

The raw data supporting the conclusions of this article will be made available by the authors, without undue reservation.

## Abbreviations

AUC, area under curve; CSS, cancer-specific survival; GSRCC, gastric signet ring cell carcinoma; LODDS, log odds of positive lymph nodes; MLNR, metastatic lymph node ratio; NPLN, number of positive lymph nodes; OS, overall survival; PLN, positive lymph nodes; PLNM, positive lymph node number; SEER, Surveillance, Epidemiology and End Results.
